# Discrimination of Alzheimer’s disease and non‐Alzheimer’s disease neurocognitive‐disordered patients by assessing CSF biomarkers in Thai population

**DOI:** 10.1002/alz.089035

**Published:** 2025-01-09

**Authors:** Witsarut Nanthasi, Chatchawan Rattanabannakit, Natthamon Wongkom, Pathitta Dujada, Atthapon Raksthaput, Sunisa Chaichanettee, Paphawadee Phoyoo, Lertchai Wachirutmangur, Vorapun Senanarong

**Affiliations:** ^1^ Faculty of Medicine Siriraj Hospital, Mahidol University, Bangkok Thailand; ^2^ Division of Neurology, Department of Medicine, Faculty of Medicine Siriraj Hospital, Mahidol University, Bangkok Thailand

## Abstract

**Background:**

CSF biomarkers including β‐amyloid (Aβ) (1‐42), phosphorylated tau (p‐tau) 181 and total tau (t‐tau) are used as ante‐mortem biomarkers to detect Alzheimer’s disease (AD) pathology in cognitively impaired patients. Decreased level of Aβ(1‐42), elevated level of p‐tau 181 and t‐tau are well established in AD. Base on CSF analysis technique and population, cut‐points are varied to distinguish between AD and non‐AD neurocognitive‐disordered patients. This study aimed to evaluate the local cut‐points of CSF biomarkers as diagnostic tools to discriminate AD and non‐AD neurocognitive‐disordered patients in Thai population.

**Methods:**

We enrolled 74 cognitively impaired patients in memory clinic at Siriraj hospital, Thailand. Participants underwent a standardized diagnostic dementia evaluation, including medical history, physical examination, neuropsychological tests using Thai Mental State Examination (TMSE), Montreal Cognitive Assessment (MoCA) and Clinical Dementia Rating Sum of Boxes score (CDR‐SB), and brain imaging. Patients were clinically diagnosed with each subtype of neurocognitive disorder without knowledge of CSF biomarker results. CSF Aβ(1‐42), p‐tau 181 and t‐tau were measure by ELISA technique. Youden index–based cut‐points of CSF biomarkers were defined using receiver operating characteristic (ROC) to distinguish between AD and non‐AD neurocognitive‐disordered patients.

**Results:**

45 (60.8%) patients were diagnosed with AD dementia and 29 (39.2%) patients were diagnosed with non‐AD neurocognitive disorders. Median values of CSF Aβ(1‐42) were significantly lower in AD group than in non‐AD group (371.0 vs. 666.0 pg/mL, p <0.001*). Median values of CSF p‐tau 181 and ratios of CSF p‐tau 181/Aβ(1‐42) were also significantly higher in AD group than in non‐AD group (p‐tau 181; 70.0 vs. 44.0 pg/mL, p = 0.002*) (p‐tau 181/Aβ(1‐42) ratio; 0.180 vs. 0.059, p <0.001*). Whereas t‐tau levels were not statistically different between groups (359.0 vs. 227.0 pg/mL, p = 0.053). Local cut‐points of CSF biomarkers to determine AD dementia were proposed as CSF Aβ(1‐42) ≤538 pg/mL (AUC 0.96, p <0.001*), CSF p‐tau 181 >56 pg/mL (AUC 0.72, p <0.001*) and p‐tau 181/Aβ(1‐42) ratio >0.094 (AUC 0.90, p <0.001*).

**Conclusion:**

The utility of CSF Aβ(1‐42) and p‐tau 181/Aβ(1‐42) ratio was notably higher in effectively distinguishing Alzheimer's disease from non‐Alzheimer's disease neurocognitive disorder within Thai population.

